# Bilateral variations in the branching of the external carotid artery: a case report

**DOI:** 10.1007/s00276-025-03653-5

**Published:** 2025-05-19

**Authors:** Robert Abbott, Sarah Ward, Maya Jodidio, George Holan, Jeremy J. Grachan

**Affiliations:** 1 Office of Education, Rutgers New Jersey Medical School, B-517 Medical Education Building 185 South Orange Avenue Newark, Newark, NJ 07103 USA; 2 Department of Medicine, Rutgers New Jersey Medical School, Newark, NJ USA; 3 Department of Surgery, Rutgers New Jersey Medical School, Newark, NJ USA

**Keywords:** External carotid artery, Posterior auricular artery, Occipital artery, Ascending pharyngeal artery, Anatomical variation

## Abstract

**Purpose:**

Various anatomical variations in the branching pattern of the external carotid arteries are known to occur with significant frequency and have been documented in published literature. The purpose of this case report is to document and discuss variations in the branching patterns of the external carotid artery as seen in an anatomical donor and determine the clinical relevance of these variations.

**Case presentation:**

A routine dissection of an 89-year-old female anatomical donor, whose cause of death was reported as acute myocardial infarction and atherosclerotic heart disease, revealed variations in the branching patterns of both external carotid arteries. Bilaterally, the common carotid arteries bifurcated at the C4 vertebral level. On the left side, an occipitoauricular trunk originated 0.5 mm superior to the common carotid artery’s bifurcation, whereas, on the right side, an occipitoauriculopharyngeal trunk branching 0.8 mm superior to the bifurcation of the common carotid artery was observed before branching into an occipitoauricular trunk and ascending pharyngeal artery.

**Conclusion:**

This case report reinforces previous publications on arterial branching patterns and the importance of imaging prior to procedures. Clinically, these variations may impact surgical approaches, endovascular procedures within the neck, and vascular pathology management.

## Introduction

The bilateral common carotid arteries bifurcate at the level of the superior border of the thyroid cartilage, between the C4 and C5 vertebral levels, into the internal and external carotid arteries [2, 10]. The external carotid arteries, anterior and lateral to the internal carotids, have multiple branching arteries that supply significant blood flow to the neck, head, and face [2, 8]. The typical order of the branches arising from the external carotid, from inferior to superior, are the superior thyroid, ascending pharyngeal, lingual, facial, occipital, posterior auricular, and a terminal bifurcation into the maxillary and the superficial temporal arteries [8].

This case report describes variations in the branching of the external carotid artery, specifically of the ascending pharyngeal, occipital, and posterior auricular arteries. The ascending pharyngeal artery commonly branches from the posterior wall of the external carotid artery and courses superiorly to supply the pharynx, middle ear, dura mater, and cranial nerves IX through XII [4, 6, 8]. The occipital artery courses posterosuperiorly to supply structures in the posterior neck and scalp [5, 6, 8]. The posterior auricular artery also arises posteriorly, above the digastric and stylohyoid muscles, to supply the parotid gland, musculature in the neck, and scalp behind and above the ear [8].

Previous research has documented numerous variations in branching of the external carotid artery [2, 4, 6, 10]. Embryologically, these variations are likely due to incomplete separation or abnormal anastomoses with other branches of the external carotid. Bergman’s Illustrated Encyclopedia of Human Anatomic Variation suggests some of the well-documented variants in the branching of the external carotid artery, including the presence of various trunks, such as an occipitoauricular trunk, a linguofacial trunk, a thyrolingual trunk, or a thyrolinguofacial trunk [9]. This case report presents unique bilateral variations in the branching patterns of the external carotid artery and discusses the clinical relevance of these variations.

## Case presentation

**Fig. 1:**
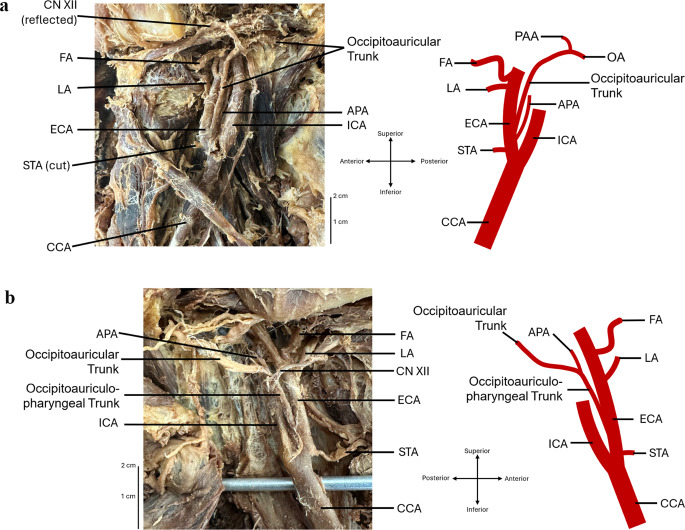
(**a**) Dissection and illustration of the left-sided variation with the occipitoauricular trunk. (**b**) Dissection and illustration of the right-sided variation with the occipitoauriculopharyngeal trunk. APA (ascending pharyngeal artery), CCA (common carotid artery), ECA (external carotid artery), FA (facial artery), ICA (internal carotid artery), LA (lingual artery), OA (occipital artery), PAA (posterior auricular artery), STA (superior thyroid artery)

During a routine dissection of an embalmed 89-year-old Caucasian female anatomical donor during the second year of medical school at Rutgers New Jersey Medical School, unique external carotid artery branching patterns were found bilaterally. The dissection of this region was guided by an internally created dissection manual provided to the students and verification of the vessels’ identity was confirmed based on the typical anatomical course and relative landmarks outlined in Gray’s Anatomy: The Anatomical Basis of Clinical Practice and other published work on variations [4–6, 8]. The anatomical donor was donated to the Rutgers Robert Wood Johnson Medical School Anatomical Association Body Program, and all ethical guidelines outlined by the program were followed throughout the study. The individual’s cause of death was reported as acute myocardial infarction and atherosclerotic heart disease. The bifurcation of the left and right common carotid arteries was found in a typical presentation at the C4 vertebral level. Bilaterally, the external carotid artery immediately branched to give off the superior thyroid arteries, coursing along their usual path toward the thyroid gland. The left side was found to have an occipitoauricular trunk (i.e., a common trunk for the occipital and posterior auricular arteries) originating 0.5 mm from the bifurcation of the common carotid artery [Fig. 1a]. Similarly, an occipitoauriculopharyngeal trunk (i.e., a common trunk for the occipital, posterior auricular, and ascending pharyngeal arteries) was found on the right side 0.8 mm above the bifurcation [Fig. 1b]. The common trunks on both sides coursed deep to the hypoglossal nerve. The left-sided 6 cm long occipitoauricular trunk coursed posteriorly towards the outer ear before splitting to give rise to the occipital artery and posterior auricular artery, with the occipital branch coursing between the splenius capitis muscle and semispinalis capitis muscle. The right-sided occipitoauriculopharyngeal trunk traveled for 2.1 cm before bifurcating into an occipitoauricular trunk and the ascending pharyngeal artery. Similar to the left side, the common trunk coursed posteriorly between the splenius capitis muscle and semispinalis capitis muscle, while the ascending pharyngeal artery continued deep to the trunk and ascended superiorly, anterior to the longus capitis muscle. Identification of the vessels was based on the descriptions of the normal course of each artery, as outlined in the introduction.

## Discussion

Existing studies have noted the prevalence of variations in the external carotid artery, including the level of bifurcation of the common carotid artery, various common trunks for its branches, and accessory branches [2, 9]. The current case study focuses on the unique branching and formation of common trunks involving the ascending pharyngeal artery, posterior auricular artery, and occipital artery. One meta-analysis of anatomical patterns of the occipital artery found that the prevalence of the occipital artery forming a common trunk with another artery was 13.91%, most commonly forming a common trunk with the ascending pharyngeal artery [5]. A less common variation is the occipital artery arising from the internal carotid artery [6, 9]. The variations most commonly seen in the ascending pharyngeal artery are its origin from the extracranial portion of the internal carotid artery or the occipital artery [4]. One study of 80 anatomical donors found that 78/80 followed the typical ascending pharyngeal artery branching pattern; the two exceptions were an abnormally high origin and the presence of two ascending pharyngeal arteries arising from one external carotid artery [2]. Additionally, this study found no significant variations in the occipital artery or the posterior auricular artery [2]. Variations in the posterior auricular artery are less discussed in the existing literature.

Well-documented variants in the branching of the external carotid artery include, most commonly, a linguofacial trunk seen in 20% of patients, followed in frequency by an occipitoauricular trunk (12.5%), a thyrolingual trunk (2.5%), and a thyrolinguofacial trunk (2.5%) [10]. The frequency of bilaterality compared to unilaterality for these abnormal branching patterns has not been widely documented in the literature, with one study noting the presence of bilateral linguofacial trunks in 10 out of 80 cases. There is a dearth of cases reported of occipitoauriculopharyngeal trunks.

The embryological process by which this abnormality may have arisen is best understood by following the normal development of the external carotid artery’s branches. One review expanded on the detailed work of Padget regarding the development of the craniofacial arteries, noting that many of the branches of the external carotid artery appear in Padget stage 5 (Carnegie stages 18–19) [1]. Throughout the formation of these branches, some anastomoses are also occurring with other developing branches [1]. Collectively, variations in the initial branching of the vessels arising from the external carotid artery, as well as other abnormalities with anastomoses and separation of developing vessels, could lead to variations such as those seen in this case report.

Knowledge of the possible variations of the external carotid artery is essential clinically due to the number of diagnostic and therapeutic procedures that require a detailed understanding of the anatomy of the external carotid artery and its branches. For example, this knowledge is critical during vascular procedures such as carotid angioplasty and extracranial-intracranial arterial bypass [10]. Furthermore, dissection of the neck is required for numerous general, vascular, maxillofacial, otolaryngeal, and plastic surgeries; knowledge of possible variations in the branching of the external carotid artery can help prevent any accidental damage [6]. Additionally, many head and neck cancers are treated with intra-arterial chemotherapy, and knowledge of any variations in arterial courses or branches is critical to ensure that the catheter is placed in the proper location and does not cause any unintentional damage to other structures [7]. The occipital artery has also been linked to other clinical correlations, such as its relationship with the greater occipital nerve and a theory that it can be a peripheral trigger of migraine headaches, its usage in revascularization procedures, and reconstructive flaps for scalp defects [5]. Lastly, the variation in the occipital artery is also significant due to its potential relevance to dural arteriovenous fistulas (DAVFs), which have been seen to commonly involve the occipital artery, and while a DAVF was not present in this patient, the anatomical variation here could further complicate the management of an occipital DAVF [3]. Collectively, this case presentation highlights the importance in radiologic imaging, such as arteriograms, to check for variations in patients before procedures.

## Conclusion

This case report presents discrete bilateral variations in the branching patterns of the external carotid arteries, including an occipitoauricular trunk on the left side and an occipitoauriculopharyngeal trunk on the right side. These unique variations, especially their presence bilaterally, add to previous literature on arterial branching variations of occipital trunks of the external carotid artery. This case study provides additional examples of vascular variations that may be useful in the evaluation, treatments, and surgical interventions involving this vessel or nearby structures.

## Data Availability

No datasets were generated or analysed during the current study.
